# Isoforms of Phosphorylated Tau as Potential Biomarkers for Alzheimer’s Disease: The Contribution of Mass Spectrometry-Based Proteomics

**DOI:** 10.3390/neurosci6020050

**Published:** 2025-06-03

**Authors:** Marco Agostini, Pietro Traldi, Mahmoud Hamdan

**Affiliations:** Istituto di Ricerca Pediatrica Città della Speranza, Corso Stati Uniti 4, 35100 Padova, Italy; m.agostini@unipd.it (M.A.); mhglaxo@gmail.com (M.H.)

**Keywords:** Alzheimer’s disease (AD), disease heterogeneity, post-translational modifications (PTMs), mass spectrometry (MS)-based proteomics, amyloid-β (Aβ), isoforms of phosphorylated tau

## Abstract

Alzheimer’s disease (AD) is a progressive neurodegenerative disorder, heterogeneous at the molecular level and characterized by diverse and complex pathological features. Such features are known to accumulate silently in the brain over years or even decades before the onset of detectable symptoms. Despite long years of intense research activities, the disease remains orphaned of either disease-modifying therapies or a specific blood test capable of predicting the disease in the pre-symptomatic stages. This disappointing outcome of such efforts can be attributed to a number of factors. One of these factors is the failure of earlier research to capture the heterogeneity of the disease. Such failure has the direct consequence of poor patient stratification, which in turn impacts negatively on the development of specific and effective therapy. The second factor is the absence of detailed and accurate information on proteins and associated post-translational modifications, which may influence the initiation and progress of the disease. Recent studies have demonstrated that the quantification of various isoforms of phosphorylated tau protein in plasma and other biofluids can be considered as potential biomarkers for the early detection of Alzheimer’s disease. Mass spectrometry-based proteomics and immunoassay-based multiplex proteomics are the two technologies in current use for probing the human proteome, both in tissues and biofluids. In the present review, we discuss the contribution of MS-based proteomics to efforts aimed at the identification and eventual characterization of the heterogeneity of the disease, and the key role of the same technique in the analysis of protein post-translational modifications associated with the disease is also discussed.

## 1. Introduction

Alzheimer’s disease (AD) is a serious neurodegenerative disorder that afflicts millions of people throughout the world. The disease results in devastating disability and diminished quality of life, and its occurrence is expected to reach epidemic proportions by 2050 if not abated. The onset of the disease is commonly diagnosed after the age of 65. AD is highly heterogeneous and is known to have a complex pathobiology. The presence of extracellular β-amyloid deposition as plaques and intracellular accumulation of hyperphosphorylated tau as neurofibrillary tangles remains the primary neuropathologic criteria for AD diagnosis. Although blood-based tests are readily available for various conditions, including cardiovascular diseases, type 2 diabetes, and some forms of cancer, Alzheimer’s disease (AD) and other neurodegenerative diseases lack an early blood-based screening test that can be used in primary care. Most of the clinical research to develop disease-modifying therapies for Alzheimer’s has focused on targeting the beta-amyloid protein. In 2021, aducanumab, an antibody targeting amyloid-beta protein deposits, received approval by the Food and Drug Administration (FDA). The approval of aducanumab and two other antibodies was mainly based on the ability of these drugs to slow cognitive decline in individuals with early Alzheimer’s disease. The initial and rather modest success of these drugs did not develop into effective disease-modifying therapies.. In other words, after decades of clinical and academic research, AD patients are still orphans of an effective disease-modifying therapy. The failure of years of research to deliver efficacious AD therapy can be partially justified by the following consideration: various studies have illustrated the varied and complex pathophysiology beyond amyloid-β (Aβ) plaques and tau neurofibrillary tangles [1,2,3]. Experience gained by the analysis of other complex diseases, such as some forms of cancer, has demonstrated that the heterogeneity of a given disease is central not only to a correct stratification of patients but also for the design of effective therapy. A recent MS-based proteomic study reported that AD has at least five subtypes [4]. These multiple subtypes, together with other heterogeneous characteristics of the disease, have to be taken into serious consideration in the design and development of future AD therapies. Another issue as important as finding disease-modifying therapies is the discovery and validation of specific, multiple AD biomarkers. Currently, there have been various attempts to go beyond Aβ and tau in the investigation of the disease. Over a number of years, proteomic meta-analyses of biofluids, including cerebrospinal fluid and serum from AD patients, have revealed thousands of differentially expressed proteins in the landscape of AD brain proteins [5]. Deeper characterization of these proteins, as well as associated post-translational modifications (PTMs), can shed some light on certain mechanisms behind the development of the disease and will certainly help in developing more efficient biomarkers for the early detection of the disease. It has to be emphasized that the use of MS-based analysis of cerebrospinal fluid (CSF) is not limited to the identification of a high number of proteins within a given sample; the same analysis can provide crucial information on the heterogeneity and pathogenic changes of the disease. The capability of MS-based proteomics to reveal the heterogeneity of AD was demonstrated in a recent study [4]. Investigation of cerebrospinal fluid proteomics in patients with Alzheimer’s disease revealed five molecular subtypes associated with specific AD genetic risk variants. A recent study assessed protein profiles and their correlation to brain microvascular dysfunction in a number of AD patients [6]. Earlier studies demonstrated the important role of brain vasculature in various neurodegenerative disorders [7,8]. Samples from both AD patients and controls were examined by liquid chromatography coupled to tandem mass spectrometry (LC-MS/MS) operated in data-independent acquisition mode (DIA). Comparing generated protein profiles in the two groups revealed enhanced protein expression in AD samples. The authors interpreted such enhanced expression as an adaptive response to compensate for cellular toxification. An example of the application of the same type of analysis to investigate AD pathogenic changes was given in a recent work [6]. Further details on these highly informative studies, together with other MS-based investigations, are discussed in more detail in the Discussion section.

**Figure 1:**
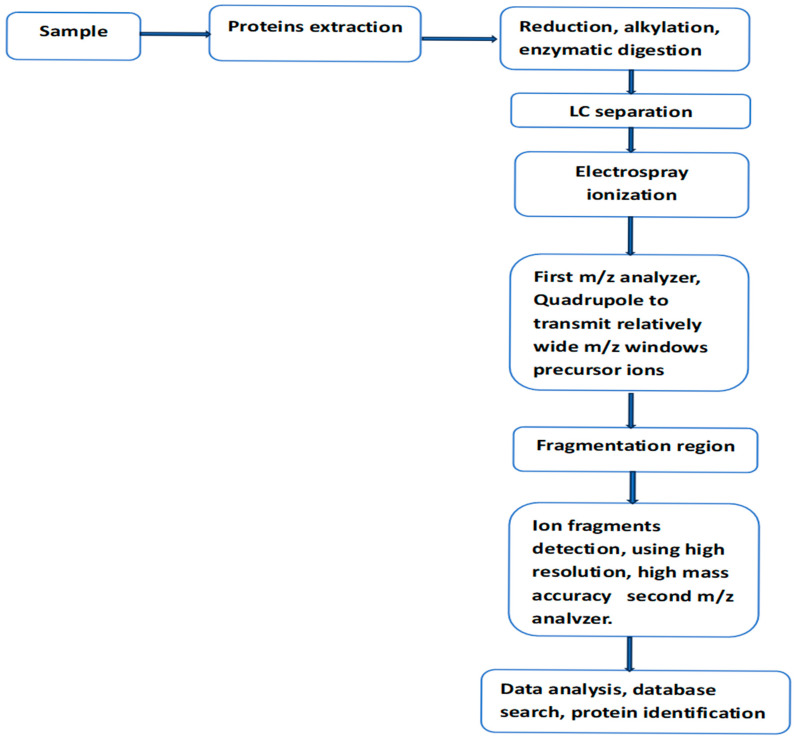
Workflow for LC-MS/MS analysis of protein mixture within a biological sample, using data-independent acquisition mode (DIA). In this mode of analysis, sequential window acquisition of all theoretical mass spectra (SWATH-MS) is used for the detection and quantification of non-labeled proteins within the analyzed sample.

For over thirty years, liquid chromatography coupled with tandem mass spectrometry (LC-MS/MS) has been the workhorse in the analysis of complex protein mixtures present in tissues or biofluids. These analyses can provide specific and accurate information on protein identity, its interaction with other proteins/ligands, and the location and identity of associated post-translational modifications (PTMs). It has to be said that the clinical value of the proteomic data generated by this technique is likely to be more valuable when combined with data sets generated by genetic, epigenetic, and transcriptional methods. The basic components of the experimental setup and the procedure for protein analysis have remained unchanged for over three decades (Figure 1) [9,10,11,12]. During the same period, a number of improvements have taken place, including the introduction of high-resolution, high-accuracy mass instruments; more powerful data acquisition modes; and the optimization of sample preparation, including protein labeling, efficient ion fragmentation methods, and more powerful software packages. These developments and their impact on MS-based proteomics have been widely discussed in recent investigations [13,14,15]. Data-independent acquisition (DIA) is one of the more recent developments for MS/MS analysis. This mode of acquisition is known to improve proteins quantification, particularly in complex biological samples, and it reduces the number of ions, which can be missed by other well established acquisition methods [15].

Sequential window acquisition of all theoretical mass spectra (SWATH-MS) was first demonstrated using a quadrupole time-of-flight instrument (Q-TOF) [16]. On the software side, the method uses ion fragmentation patterns already present in existing spectral libraries to interpret MS/MS spectra generated by DIA. In this mode of data acquisition, the quadrupole transmits to the collision region a relatively wide consecutive precursor ion window. This scan procedure usually covers the m/z range sufficiently to allow the fragmentation of most peptides in a complex protein digest. In this method of analysis, only highly sophisticated and rather complex software can handle the complexity of the generated MS/MS spectra.

In the course of the present discussion, two other methods used for protein determination in biofluids will be cited: SomaScan and PEA. Over the last ten years, SomaScan has emerged as a powerful technology in the field of biomarker discovery. The SomaScan assay is a highly multiplexed proteomic method that uses SOMAmer reagents to detect proteins in various biological samples. This method is a high-throughput, aptamer-based proteomics assay capable of the simultaneous measurement of thousands of proteins over a wide range of endogenous concentrations using relatively low sample volumes. The proteomic coverage of this technology has increased from 800 proteins over 15 years ago to about 11,000 at the present time. Technical details, advantages, and limitations of this method have been given in various articles [17,18,19]. Proximity extension assay (PEA) is an immunohistochemical tool that uses two or more DNA-tagged aptamers or antibodies binding in close proximity to the same protein or protein complex. Amplification by PCR or isothermal methods and hybridization of a labeled probe to its DNA target generates a signal that enables the sensitive and robust detection of proteins, protein modifications, or protein–protein interactions [20].

## 2. Discussion

### 2.1. Mass Spectrometry-Based Investigation of AD Heterogeneity

Identification and eventual understanding of the heterogeneity of a given disease is considered a precondition for correct patient stratification, the development of effective disease-modifying therapies, and the discovery of specific disease biomarkers. Disease heterogeneity has been extensively studied in various forms of cancer, in studies that contributed to a better understanding of tumor resistance and the development of more effective targeted therapies. Till very recently, AD has been considered as a disease composed of a single sub-population, which explains the persistence in considering the pathological hallmarks of AD to be the two proteins, β-amyloid (Aβ) and tau. Over the last five years, a number of investigations have clearly demonstrated that at the molecular level, the disease is biologically heterogeneous, composed of at least five subtypes [4,21,22]. Before discussing these and other emerging information regarding the characterization of the various components of AD heterogeneity, the following consideration might be helpful. It can be argued that the search for disease-modifying therapies and/or multiple specific biomarkers without full knowledge of AD heterogeneity can be compared to the search for a black object in a dark tunnel. The long experience in dealing with biologically complex diseases, such as certain forms of cancer, has demonstrated that a better understanding of the heterogeneity of these diseases is central to the understanding of their resistance to therapy and the failure to develop effective drugs against these diseases. It is also reasonable to argue that deciphering AD heterogeneity is not only crucial for therapy development but also a major step in the quest for personalized medicine. To underline the contribution of MS-based proteomics to efforts aimed at understanding the heterogeneity of AD, a number of recent investigations are here discussed. In one of these studies [4], the authors used MS-based proteomics to assess the expression levels of over 1000 proteins present in cerebrospinal fluid derived from AD patients (over 400) and controls (187). Data generated by this study supported the findings of two earlier studies conducted by the same research group [21,22]. According to the same study, alterations in protein levels were associated with distinct molecular processes and five different subtypes: neuronal hyperplasticity, innate immune activation, RNA dysregulation, choroid plexus dysfunction, and blood–brain barrier impairment. Furthermore, each subtype was related to specific AD genetic risk variants. According to the same study, the identified subtypes also differed in clinical outcomes, survival times, and anatomical patterns of brain atrophy. The study we are discussing has furnished valuable information regarding the different AD subtypes and their role in the heterogeneity of the disease. That said, the same study has a number of limitations, some of which have been pointed out by the authors in their study. For example, the assignment of AD patients was based on disease biomarkers, strongly associated with the pathology of the disease; however, for some patients, the same biomarkers could not guarantee the accuracy of such assignment (definition). Another limitation of the same study was the non-availability of samples from clinical trials to establish whether the detected AD subtypes would respond differently to treatment. Another limitation, which has not been considered by the authors, is related to the mode of data acquisition in the LC-MS/MS analysis; the authors used data-dependent acquisition mode (DDA). In this mode of acquisition, a number of low-intensity ions are not transmitted by the first analyzer (MS1) to the fragmentation region (an effect known as missing masses). In other words, some protein alterations were not captured by this mode of analysis. This effect is strongly attenuated in data-independent acquisition mode (DIA), where MS1 is operated in a fashion to transmit most of the ions to the collision region. A multilayer network approach has been used to assess AD heterogeneity and its impact on the diagnosis and progression of the disease [23]. In their study, the authors tested a multi-dimensional network framework applied to 490 subjects, including cognitively normal, mild cognitive impairment, and AD patients. Based on structural magnetic resonance imaging, amyloid-β positron emission tomography, cerebrospinal fluid, cognition, and genetics data, subject similarity networks were constructed. This study reported the identification of AD subtypes that shared similar multidisciplinary profiles of neurological, cognitive, pathological, and genetic information.

An interesting study to probe the heterogeneous nature of AD was recently reported [24]. In their study, the authors investigated the correlation between spatially remote neurophysiological events (functional connectivity). This study involved a total of 82 AD patients and 50 controls, comprising men and women aged between 55 to 90 years. The authors used functional magnetic resonance imaging (fMRI), a technique capable of shedding some light on the interaction between different regions of the brain [25]. One of the conclusions in reference [24] is the identification of two AD subtypes, described as benign and malignant subtypes. The authors attributed the difference between the two subtypes to different degrees of disruption in two sets of functional connections. According to the same study, the malignant subtype exhibited extensive functional connectivity loss, while the benign subtype showed mild impairments within the limbic system and its associated resting-state networks. The authors of the same article observed that subjects suffering from the benign subtype demonstrated much higher functional connectivity compared to those suffering from the malignant form of the disease; furthermore, the first category exhibited improvement after a certain age. It can be said that data presented in the same article not only contribute to attempts to understand the heterogeneity of AD, but also underlines possible differences in functional connectivity among different subtypes of the disease. That said, and considering the very limited number of both AD patients and controls, the above findings have to be considered as indications that have to be verified on a much higher number of both patients and controls. 

### 2.2. Protein Post-Translational Modifications in Alzheimer’s Disease

There is clear evidence that protein post-translational modifications (PTMs) alter the charge and hydrophobicity of a protein, in turn inducing structural changes that influence protein function, protein–protein interactions, and protein aggregation [26]. Practically most proteins, including the “intrinsically disordered” or “natively unfolded” proteins that tend to aggregate in neurodegenerative diseases, are susceptible to PTMs [27]. Natively unfolded proteins do not have a defined three-dimensional structure, which renders them highly exposed to numerous PTMs. Tau is an example of a natively unfolded protein that can experience various PTMs. Tau is a microtubule-associated protein encoded by the *MAPT* gene, which is located on chromosome 17q21 and contains 16 exons. The primary structure of tau consists of an N-terminal projection domain, a proline-rich region, a repeat region, and a C-terminal domain [28] (Figure 1). Tau has six different isoforms in the human brain that differ from each other in the number of N-terminal inserts [29], and the two PTMs, which are known to influence tau aggregation, are considered below.

#### 2.2.1. Tau Phosphorylation

**Figure 2:**
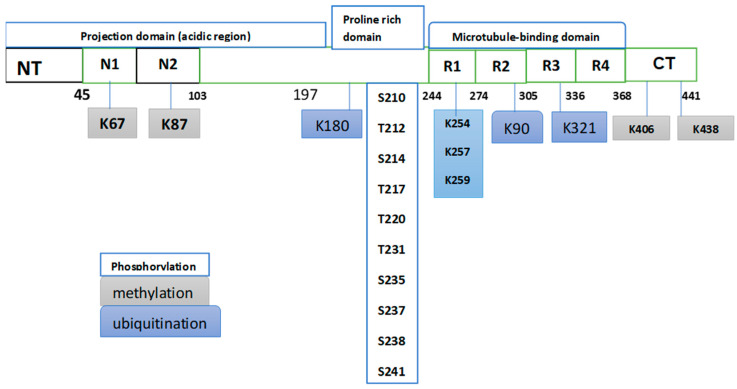
Partial representation of some PTMs occurring in tau from postmortem AD brains. This partial presentation shows that phosphorylation is the dominant PTM. The original Fig. in Ref. [34] listed 50 phosphorylation sites distributed heterogeneously in the tau sequence, with the most abundant p-sites clustering within the proline-rich domain. The same Fig. showed sites of methylation and ubiquitination. Phosphorylation involves two amino acids, serine (S) and threonine (T), while methylation and ubiquitination involve lysine (K).

Proteins’ post-translational modifications (PTMs) are central in the regulation of many biological functions, and their dysregulation is frequently linked to various diseases, which explains why such regulation is an attractive target for drug discovery and development. The current literature clearly demonstrates the role of certain PTMs in various serious diseases; for example, ubiquitination of the androgen receptor (AR) is recognized as a crucial regulatory factor in the development of various tumors, including prostate cancer [30]. Protein phosphorylation is one of the PTMs that is drawing intense pharmacological attention in the search for drug targets, with interest ranging from oncology to neurodegeneration [31]. It is interesting to note that tau protein hyperphosphorylation was the first pathological post-translational modification to be linked to Alzheimer’s disease (AD) [32]. It can be said that tau phosphorylation can play a dual role in its interaction with AD. Part of the current research effort is focused on the potential role of this modification as a specific biomarker of the disease, while a different line within the same research is investigating this modification as part of new therapeutic strategies. To assess the role of this modification in relation to AD, a number of recent investigations dealing with both roles of this modification are considered. Phosphorylation is the main modification, which occupies more than 50 sites. Other significant PTMs include methylation, acetylation, and ubiquitination [33,34]. There are a number of recent works dealing with the potential of phosphorylated tau as a biomarker for AD and the challenges on the way to such an objective. One of the relevant arguments raised in some of these works is a comparison of performance between singly and multiply phosphorylated tau, in particular regarding the specificity of such performance. Figure 2 gives a partial representation of some PTMs of tau.

In a relatively recent study, the authors developed two immunoassays for the detection of phosphorylated tau in CSF and plasma samples [35]. These assays were used to measure CSF and plasma p-tau181&231, p-tau217&231, p-tau181, p-tau217, and p-tau231 levels in two cohorts across the AD continuum and in frontotemporal dementia (FTD) patients.This study concluded that the level of p-tau181 and 231 within the CSF samples is a better AD biomarker than those observed in plasma samples. On the other hand, the same article proposed p-tau217 and 231 as promising biomarkers in both CSF and plasma samples. Given the recent publication of this article and its main conclusions, the following observation can be made: the detection of p-tau in plasma samples is relevant to research efforts to discover and develop blood-based biomarkers for AD. The same study has a number of limitations. As mentioned above, tau can be phosphorylated at over 50 sites, yet the authors do not explain why the three phosphorylation sites monitored in their study should be considered better biomarkers than the rest of the phosphorylation sites. It is still unclear whether the multiplicity of phosphorylation is significantly linked to the progression of AD. In other words, can the number and sites of phosphorylation be linked to the phase of development of the disease? Without a convincing answer to such questions, it is still premature to claim that multiply phosphorylated tau is a better biomarker than singly phosphorylated tau.

The current literature indicates that MS-based studies for the simultaneous quantification of different isoforms of p-tau in plasma are still very limited. One of these studies used immunoprecipitation in combination with LC-MS/MS for the simultaneous quantification of six p-tau isoforms in over 200 plasma samples obtained from AD patients. The main objective of the study was the assessment of a possible correlation between the various isoforms and AD-related brain changes. The investigated isoforms were phosphorylated at threonine in positions p-tau181, 199, 202, 205, 217, and 231. This study reported that quantification of p-tau205, 217, and 231 involved the plasma tau isoforms that best reflected AD pathological changes. Some of the results generated by this investigation coincide with the results of earlier studies. For example, two MS-based investigations were in agreement that site-specific phosphorylation of tau seems to occur at different periods (stages) of the disease progression [36,37,38,39]. Other results indicated that the plasma level of p-tau231 could indicate early changes in preclinical AD, earlier than the manifestation of amyloid PET abnormalities; furthermore, the level of the same isoform did not experience further increase in subjects with more serious symptoms of the disease [36,38]. This result was tentatively attributed to a strong correlation between p-tau231 levels and amyloid pathology, which increases with the early accumulation of Aβ and flattens in the later stages [39]. According to the same study, the level of non-phosphorylated tau species tau195–209 and tau212–221 did not change significantly along the AD continuum. Accurate quantification of these peptides in plasma is known to be negatively influenced by the contribution of peripheral tau, which tends to mask the measurement of brain-specific tau [40]. 

#### 2.2.2. Methylated Tau

Methylation is considered a key regulator of tau protein in Alzheimer’s disease. Tau protein can experience mono- or di-methylation. This PTM occurs at several lysine and a few arginine residues through the action of two enzymes, lysine methyl transferases or arginine methyl transferases [41]. Under physiological conditions, tau was found to be methylated at 11 Lys sites [42]. This PTM directly impacts tau biology by lowering its aggregation propensity. Lys methylation may also enhance tau’s capability to interact with several known binding partners [43]. An early work also confirmed that several phosphorylation and methylation sites in tau are present in close proximity, which may alter the occurrence of both modifications. Tau phosphorylation at Ser262 is known to induce tau aggregation, and phosphorylated tau fragments R1-pS262 directly result in neuropathological changes [44]. Present information on tau methylation in AD indicates that methylation occurs in both normal tau as well as its pathological form as paired helical filaments (PHFs), and the arginine residues R126, R155, and R349 are known to be mono-methylated in both normal and pathological tau [45]. The role of methylated tau in AD is still under investigation. The use of MS-based proteomics can furnish relevant information for such a role. To identify Lys-residue modifications associated with normal tau function, soluble tau proteins isolated from four cognitively normal human brains were examined by LC-MS/MS [42]. This investigation furnished a number of relevant findings regarding soluble tau’s methylation sites, their distribution, and likely cross-talk with other modifications. This study reported that mono- and di- methylation greatly suppressed tau aggregation propensity by depressing the nucleation rate, inhibiting the elongation rate, and destabilizing mature filaments. The same study proposed that multiple tau PTMs, including phosphorylation and methylation, act in concert to affect both normal and pathological functions of tau.

### 2.3. Plasma-Based Biomarkers for the Diagnosis of Alzheimer’s Disease

**Figure 3:**
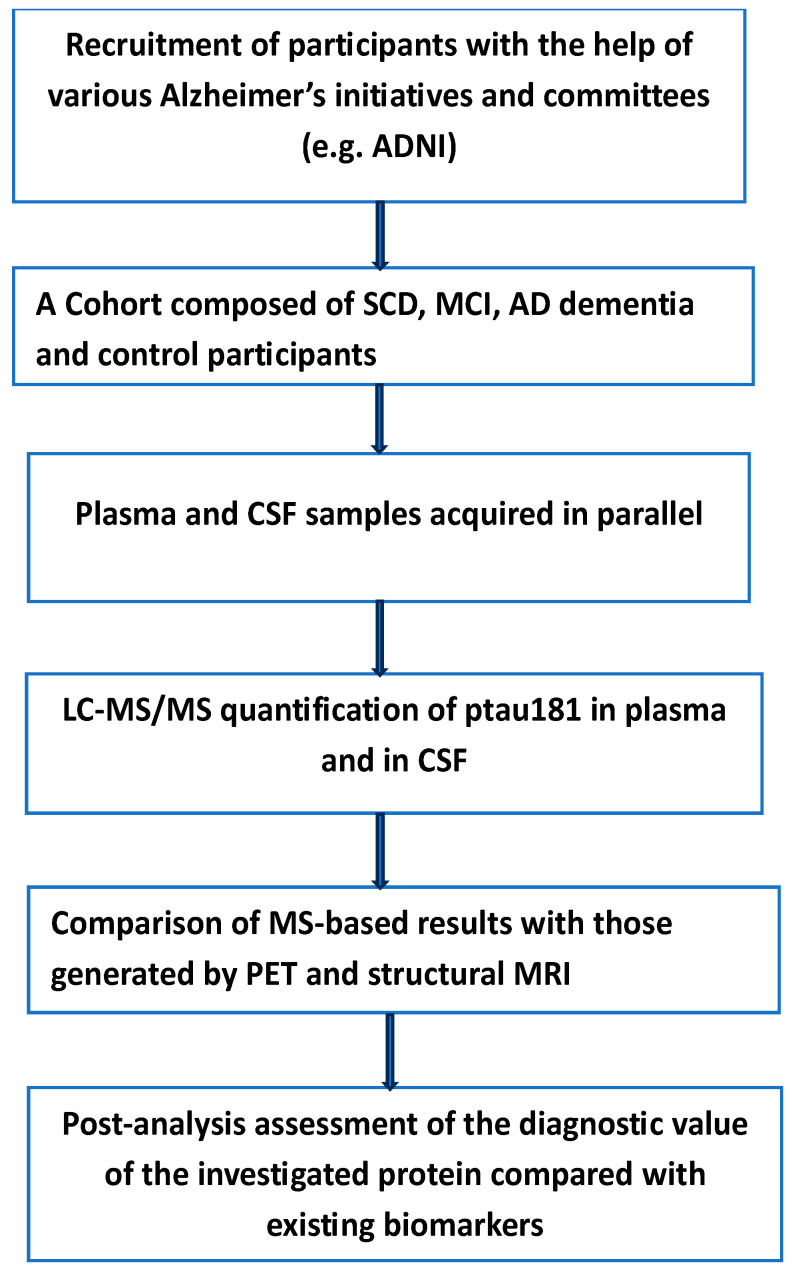
The main steps in the quantitative determination of p-tau181 in both plasma and CSF of participants with various degrees of AD. Subjective cognitive decline (SCD), mild cognitive impairment (MCI). Measurable variation in the level of this protein can reflect the extent of the disease in both testing and validation cohorts. ADNI (Alzheimer’s Disease Neuroimaging Initiative).

Plasma-based tests are readily available for various serious diseases, including cardiovascular disease, type 2 diabetes, and a number of cancer types. Alzheimer’s disease (AD) and other neurodegenerative diseases are still orphans of an early blood-based screening test that can be used in primary care. Currently, the gold standard biomarkers for Alzheimer’s disease diagnosis in clinical practice are CSF amyloid-β (Aβ), phosphorylated tau, and total tau, supported by advanced neuroimaging methods and positron emission tomography (PET). Invasiveness, cost, and limited accessibility render their clinical practice rather limited. These limitations, together with an accelerated approval by the FDA of two highly promising monoclonal antibodies (aducanumab and lecanemab) as therapy for AD, have encouraged the search for a new class of biomarkers. There is a diffused scientific opinion that suggests that if these potentially disease-modifying anti-amyloid therapies become widely available for use in clinical practice, it will become even more urgent to establish an early and accurate diagnosis of AD [46,47,48]. Over the last few years, research efforts have focused on the development of blood-based biomarkers, which can be less invasive and have higher predictive efficacy than existing biomarkers. Compared to CSF and PET analyses, blood-based analysis is non-invasive, more accessible, less expensive, and allows serial sampling, which facilitates continuous updating of the state of the disease. Currently, there are a number of plasma-based biomarkers under investigation in various clinical cohorts (see Table 1 and Figure 3). The works listed in Table 1 support the following observations: (a) The performance of APOE as a potential AD biomarker was highly enhanced through its combination with plasma Aβ42/40 ratio and the age of the patient [49]. The same combination showed good accuracy in diagnosing brain amyloidosis, using LC-MS analysis [50]. (b) In the earlier stages of the AD pathological continuum, plasma p-tau217 was found to increase earlier than other isoforms [51,52]; the same isoform was found to have good accuracy in distinguishing Aβ-PET+ from Aβ-PET–cognitively unimpaired individuals [51,53]. (c) The plasma Aβ42/40 ratio was found to have a stronger correlation with brain Aβ burden and better diagnostic and prediction accuracy than either Aβ42 or Aβ40 alone [54,55]. Until very recently, measurement of this ratio was limited to CSF samples; however, the emergence of highly sensitive assays has allowed the measurement of plasma low levels of this ratio. Plasma YKL-40 has been strongly linked to non-AD neurodegenerative diseases rather than with AD dementia [56]. CSF YKL40 is classified as a marker of neuroinflammation, has been reported to increase with Aβ accumulation [57], and could differentiate between healthy controls and AD patients [58]. It is relevant to point out that the majority of the works cited in Table 1 are in agreement that none of the individual markers cited in the same table had the desired performance as biomarkers for the early detection of AD or the ability to differentiate it from other neurodegenerative diseases.

To evaluate the clinical value of plasma p-Tau181 to predict Alzheimer’s disease, the authors investigated the clinical applicability of this biomarker in the screening of patients in primary care centers and memory clinics with a suspected high risk of developing AD dementia [70]. Three large independent cohorts with more than 2000 patients were involved. This relatively high number of participants allowed access to more than 1800 CSF/plasma-paired samples obtained on the same day. The experimental details and methods employed have been given in the original article, and we see no benefit in repeating them here. Instead, we want to underline the findings and limitations of the same study. According to the authors, the main finding of their study was the ability of plasma p-Tau181 to predict the transition from mild cognitive impairment (MCI) to AD dementia. Furthermore, subjects with MCI who moved to AD dementia exhibited higher values of plasma p-Tau181 levels than non-mover MCI.

The results of the same study showed that plasma p-tau181 correlated with CSF p-tau181 in both MCI and AD dementia, a result in agreement with earlier studies [71,72]. This was not the case for subjective cognitive decline (SCD) participants. This negative result was interpreted by the authors as the result of too early a stage of the SCD phase in the disease continuum to be translated into sufficient levels of p-tau181 in the plasma. The authors of this well-designed study pointed out a number of limitations: Due to difficulties in recruiting participants with SCD together with ethical restrictions on performing a lumbar puncture, most of the SCD participants came from research studies, which could contribute to unexpected selection bias. These complications resulted in a low number of SCD participants compared to numbers in other groups. Furthermore, due to the healthier condition of this group of participants, the number of Aβ (+) patients was low. According to the authors, the classification system, amyloid–tau–neurodegeneration (AT (N)) [73], was not used because of its high costs and its invasiveness; therefore, they applied CSF as the gold standard reference.

The last few years have witnessed a substantial increase in the use of MS-based proteomics in the investigation of various biofluids, including cerebrospinal fluid. For example, cerebrospinal fluid and plasma samples provided by an equal number of both AD patients and controls were analyzed by three different proteomic platforms [74,75]: untargeted tandem mass tag–mass spectrometry (TMT-MS), proximity extension assay (PEA), and a modified aptamer-based assay. Mass spectrometry measurements were performed using isobaric tandem mass tags (TMT) with pre-fractionation, including both with and without prior depletion of highly abundant proteins in each fluid. For PEA analyses, the study used all thirteen qPCR-based human biomarker panels encompassing 1196 protein assays. For aptamer-based analyses, the authors used the SomaScan assay, which provides 7288 SOMAmers targeting 6596 unique proteins. The authors reported alterations in protein levels in both CSF and plasma samples. It is interesting to note that many proteins altered in AD CSF were found to be altered in the opposite direction in plasma, including important members of AD brain co-expression modules. Several isoforms of p-tau, such as p-tau181, p-tau217, and p-tau231, can be detected in plasma with high accuracy; these entities have been the subject of various investigations and some clinical trials to explore their potential as blood-based markers for an early and accurate diagnosis of AD [76,77,78]. The main objective of most of these studies was to establish the specificity and accuracy of these plasma biomarkers for the early diagnosis of AD and their capability to differentiate between AD and other neurodegenerative disorders. In one of these studies, the accuracy of p-tau217 in distinguishing AD from other neurodegenerative diseases was assessed [51]. Plasma samples were obtained from three cohorts from three different countries, comprising AD patients, mildly cognitively impaired, and cognitively unimpaired participants. In another cohort study [79], the authors assessed how early in the course of Alzheimer’s disease plasma levels of P-tau217 begin to change; these levels were compared with the well-established cerebrospinal fluid and positron emission tomography (PET) tau biomarkers for the early diagnosis of AD. Despite a limited number of participants, about 1400 in the first study and about 500 in the second, the following conclusions were made: Plasma levels of p-tau217 can be used to discriminate between AD and other neurodegenerative diseases with higher accuracy than either p-tau isoforms or well-established MRI-based biomarkers [51]. An increase in p-tau217 was evident in the early preclinical stages of the disease; interestingly, such an increase manifested prior to the detection of insoluble tau aggregates by means of tau-PET [80].

As is the case with investigations that explore new areas of research, this investigation has a number of limitations, which have been pointed out by the same authors: The number of participants was rather low. The lack of longitudinal plasma p-tau217 data prevented more accurate comparison with a small number of participants with longitudinal tau-PET scans. Another limitation is that plasma P-tau217 levels were below the detection limit of the assay for some of the cases. For some of the investigated participants, plasma p-tau217 levels were below the detection limit of the assay used for this investigation. Blood-based biomarkers can be monitored longitudinally, a characteristic associated with a non-invasive sampling method compared to CSF or tissue sampling. This highly attractive characteristic was used to assess plasma phosphorylated tau181 (p-tau181) as a progression biomarker in Alzheimer’s disease [79] in a cohort of 1184 participants, including 403 cognitively normal patients, 560 patients with mild cognitive impairment, and 221 with AD dementia from the Alzheimer’s Disease Neuroimaging Initiative (ADNI). Plasma p-tau181 levels were measured using the single-molecule array (Simoa) technique with an in-house assay. The authors reported that plasma p-tau181 levels increased over time with disease progression. Compared to cognitively normal participants, this study showed that plasma p-tau181 levels increased at baseline in patients with mild cognitive impairment and dementia. Furthermore, increasing levels of plasma p-tau181 over time were identified in the preclinical Aβ-positive cognitively normal, prodromal Aβ-positive MCI, and dementia Aβ-positive dementia stages of Alzheimer’s disease. In an earlier study, longitudinal plasma p-tau181 levels were used to predict AD pathology several years before post-mortem results [81]. To underline the role of MS-based proteomics in the analysis of AD biomarkers in both CSF and plasma, a list of investigations and the scope of analyses are given in Table 2.

## 3. Future Perspectives and Conclusions

It is well documented that molecular alterations associated with AD start in the preclinical stage, when the clinical symptoms are almost negligible. The neuropathological confirmation of amyloid-β (Aβ) plaques and tau neurofibrillary tangles (NFT) remains the gold standard for a definitive diagnosis of Alzheimer’s disease. It is alarming to state that after decades of research, AD patients are still waiting for disease-modifying therapies and for biomarkers that are specific, sensitive, and capable of AD identification in its earliest stages. The current literature on AD and on emerging therapies suggests that, once these disease-modifying therapies become available, the biggest challenge facing AD clinical practice is the identification of patients with an elevated risk of developing AD dementia and eligible for the disease-modifying treatment. In the present text, we discussed some of the difficulties that AD research had to resolve, including understanding the disease heterogeneity, the role of protein PTMs in the search for effective biomarkers, and disease-modifying therapies. Recent availability of technologies and assays sensitive enough to detect low-level modified proteins associated with AD in plasma and other biofluids has accelerated the search for new biomarkers. These research activities are still in their early stages; however, there is sufficient evidence to support a key future role of these analyses in the search for efficient biomarkers for AD screening and for the development of innovative therapies. Shifting from CSF analysis to blood-based analysis is an important step, which will facilitate more frequent use of longitudinal clinical trials essential for a disease known for its progression over time. Despite substantial progress in AD research, both regarding biomarkers for early detection of AD and potentially promising disease-modifying therapies, there are still a number of issues to be addressed to consolidate such progress: (a) Detection of various isoforms of p-tau in plasma is the focus of intense research activities aimed at the identification of more specific AD biomarkers, it can be said, that PTMs other than phosphorylation have only come into focus recently and are still understudied. Tau methylation can compete with other PTMs such as acetylation and ubiquitination, where the state of PTM at these sites impacts the function and stability of tau. The present literature suggests that this important PTM is not receiving the attention it deserves. (b) There is strong evidence that AD is a heterogeneous disease, composed of at least five subtypes. Existing experience accumulated with other serious diseases (such as some forms of cancer) suggests that the efficacy of a given therapy is strongly related to an accurate identification of the subtype that the therapy is targeting. There is still further research to be done to allow a better understanding of both the structure and the biology of AD’s heterogeneity. (c) There is still a gap between new findings in AD research and their implementation at the clinical level. For example, the use of p-tau isoform levels in plasma to predict Alzheimer’s disease pathology has been demonstrated by various research groups, yet there is a paucity of large (involving thousands of participants) clinical trials to test or validate this finding. Of course, it is not always easy or possible to recruit the desired number of participants; however, we think more efforts in this direction are warranted.

## Figures and Tables

**Table 1:** Some potential plasma and CSF biomarkers for AD screening under investigation in a number of clinical cohorts.

Potential AD Biomarkers Under Investigation	Platforms in Current Use for AD Plasma and CSF Analysis
Aβ42/40 ratio [59,60,61] Neurofilament light chain (NFL) [59,62] Glial fibrillary acidic protein (GFAP [63,64,65] p-tau isoforms (ptau181, 217, 231) [66,67,68] Apolipoprotein E (ApoE) [69] Chitinase-3-like protein 1 [YKL-40]) [58]	Electrochemiluminescence (ECL)-based methods Immunoprecipitation-LC-MS/MS Single-molecule array (Simoa) Enzyme-linked immunosorbent assay (ELISA) Sandwich ELISAs by Multimer detection system (MDS) Immunomagnetic reduction assay (IMR) Immuno-infrared sensor

**Table 2:** Examples of MS-based investigations of various AD biomarkers in cerebrospinal fluid and in plasma.

Reference	Scope of Investigation
Ref. [4] Cerebrospinal fluid proteomics in patients with Alzheimer’s disease reveals five molecular subtypes with distinct genetic risk profiles.	Defining AD molecular subtypes using mass spectrometry-based proteomics in cerebrospinal fluid.
Ref. [36] Mass spectrometric simultaneous quantification of tau species in plasma shows differential associations with amyloid and tau pathologies.	Simultaneous quantification of six p-tau isoforms in plasma and their role as biomarkers for AD.
Ref. [82] Mass spectrometry-based methods for robust measurement of Alzheimer’s disease biomarkers in biological fluids.	Investigation of AD biomarkers that can be reliably measured in CSF and plasma.
Ref. [83] Characterizing Alzheimer’s disease through integrative NMR- and LC-MS-based metabolomics.	Investigation of blood samples and extracellular vesicle metabolites to gain some insights into the pathological mechanisms of AD.
Ref. [84] Development and evaluation of a multiplexed mass spectrometry-based assay for measuring candidate peptide biomarkers in the Alzheimer’s Disease Neuroimaging Initiative.	Evaluation of a multiplexed MS-based approach for the qualification of candidate AD biomarkers in CSF samples from the AD Neuroimaging Initiative.
Ref. [85] Neuroproteomics chip-based mass spectrometry and other techniques for Alzheimer’s disease biomarkers update.	The relevance of mass spectrometry for the identification of peptides and proteins as discriminating biomarkers for AD.
Ref. [86] Mass spectrometry-based method to quantify in parallel tau and amyloid β 1–42 in CSF for the diagnosis of Alzheimer’s disease.	Development of a mass-spectrometry-based method for a parallel quantification of tau and Aβ42 in CSF.

## Data Availability

Not applicable.

## Abbreviations

AD	Alzheimer’s disease
Aβ	amyloid-β
PTMs	post-translational modifications,
LC-MS/MS	liquid chromatography-tandem mass spectrometry
PEA	proximity extension assay
FTD	frontotemporal dementia
SWATH-MS	Sequential window acquisition of all theoretical mass spectra
CSF	Cerebrospinal fluid
(AT(N))	Amyloid-tau-neurodegeneration
DMN	Default mode network (DMN)
AR	Androgen receptor (AR)
PET	Positron emission tomography
MCI	Mild cognitive impairment
SCD	Subjective cognitive decline
TMT-MS	tandem mass tag mass spectrometry
ADNI	Alzheimer’s Disease Neuroimaging Initiative
Simoa	Single Molecule array
NFT	Neurofibrillary tangles

## References

[1] Johnson E.C.B., Carter E.K., Dammer E.B., Duong D.M., Gerasimov E.S., Liu Y., Liu J., Betarbet R., Ping L., Yin L. (2022). Large-scale deep multi-layer analysis of Alzheimer’s disease brain reveals strong proteomic disease-related changes not observed at the RNA level. Nat. Neurosci..

[2] Johnson E.C.B., Dammer E.B., Duong D.M., Ping L., Zhou M., Yin L., Higginbotham L.A., Guajardo A., White B., Troncoso J.C. (2020). Large-scale proteomic analysis of Alzheimer’s disease brain and cerebrospinal fluid reveals early changes in energy metabolism associated with microglia and astrocyte activation. Nat. Med..

[3] Beckmann N.D., Lin W.-J., Wang M., Charney A.W., Wang P., Cohain A.T., Ma W., Wang Y.-C., Jiang C., Audrain M. (2020). Multiscale causal networks identify VGF as a key regulator of Alzheimer’s disease. Nat. Commun..

[4] Tijms B.M., Vromen E.M., Mjaavatten O., Holstege H., Reus L.M., van der Lee S., Wesenhagen K.E.J., Lorenzini L., Vermunt L., Venkatraghavan V. (2024). Cerebrospinal fluid proteomics in patients with Alzheimer’s disease reveals five molecular subtypes with distinct genetic risk profiles. Nat. Aging.

[5] Bai B., Vanderwall D., Li Y., Wang X., Poudel S., Wang H., Dey K.K., Chen P.-C., Yang K., Peng J. (2021). Proteomic landscape of Alzheimer’s Disease: Novel insights into pathogenesis and biomarker discovery. Mol. Neurodegener..

[6] Erickson M.A., Johnson R.S., Damodarasamy M., MacCoss M.J., Keene C.D., Banks W.A., Reed M.J. (2024). Data-independent acquisition proteomic analysis of the brain microvasculature in Alzheimer’s disease identifies major pathways of dysfunction and upregulation of cytoprotective responses. Fluids Barriers CNS.

[7] Reed M.J., Damodarasamy M., Banks W.A. (2019). The extracellular matrix of the blood-brain barrier: Structural and functional roles in health, aging, and Alzheimer’s disease. Tissue Barriers.

[8] Sweeney M.D., Kisler K., Montagne A., Toga A.W., Zlokovic B.V. (2018). The role of brain vasculature in neurodegenerative disorders. Nat. Neurosci..

[9] Yates J.R., McCormack A.L., Schieltz D., Carmack E., Link A. (1997). Direct analysis of protein mixtures by tandem mass spectrometry. J. Protein Chem..

[10] Aebersold R., Mann M. (2016). Mass-spectrometric exploration of proteome structure and function. Nature.

[11] Ye Z., Vakhrushev S.Y. (2021). The role of data-independent acquisition for glycoproteomics. Mol. Cell Proteom..

[12] Lin C.-H., Krisp C., Packer N.H., Molloy M.P. (2018). Development of a data independent acquisition mass spectrometry workflow to enable glycopeptide analysis without predefined glycan compositional knowledge. J. Proteom..

[13] Birhanu A.G. (2023). Mass spectrometry-based proteomics as an emerging tool in clinical laboratories. Clin. Proteom..

[14] Zhang B., Bassani M. (2023). Current perspectives on mass spectrometry-based immunopeptidomics: The computational angle to tumor antigen discovery. Immunother. Cancer.

[15] Fröhlich K., Fahrner M., Brombacher E., Seredynska A., Maldacker M., Kreutz C., Schmidt A., Schilling O. (2024). Data-Independent Acquisition: A Milestone and Prospect in Clinical Mass Spectrometry–Based Proteomics. Mol. Cell Proteom..

[16] Gillet L.C., Navarro P., Tate S., Rost H., Selevsek N., Reiter L., Bonner R., Aebersold R. (2012). Targeted data extraction of the MS/MS spectra generated by data-independent acquisition: A new concept for consistent and accurate proteome analysis. Mol. Cell Proteom..

[17] Candia J., Daya G.N., Tanaka T., Luigi Ferrucci L., Keenan A., Walker K.A. (2022). Assessment of variability in the plasma 7k SomaScan proteomics assay. Sci. Rep..

[18] Candia J., Antoni G., Delgado-Peraza F., Shehadeh N., Tanaka T., Moaddel R., Walker K.A., Ferrucci L. (2024). Variability of 7K and 11K SomaScan Plasma Proteomics Assays. J. Proteome Res..

[19] Timsina J., Gomez-Fonseca D., Wang L., Do A., Western D., Alvarez I., Aguilar M., Pastor P., Henson R.L., Herries E. (2022). analysis of Alzheimer’s disease Cerebrospinal fluid biomarkers measurement by multiplex SOMAscan platform and immunoassay-based approach. J. Alzheimers Dis..

[20] Arioz B.I., Cotuk A., Yaka E.C., Genc S. (2024). Proximity extension assay-based proteomics studies in neurodegenerative disorders and multiple sclerosis. Eur. J. Neurosci..

[21] Tijms B.M., Gobom J., Reus L., Jansen I., Hong S., Debritic V., Kilpert F., Ten Kate M., Barkhof F., Tsolaki M. (2020). Pathophysiological subtypes of Alzheimer’s disease based on cerebrospinal fluid proteomics. Brain.

[22] Tijms B.M., Gobom J., Teunissen C., Dobricic V., Tsolaki M., Verhey F., Popp J., Martinez-Lage P., Vandenberghe R., Lleó A. (2021). CSF Proteomic Alzheimer’s Disease-Predictive Subtypes in Cognitively Intact Amyloid Negative Individuals. Proteomes.

[23] Avelar-Pereira B., Belloy M.E., O’Hara R., Hadi Hosseini S.M. (2023). Decoding the heterogeneity of Alzheimer’s disease diagnosis and progression using multilayer networks. Mol. Psychiatry.

[24] Sheng J., Xin Y., Zhang Q., Yang Z., Wang L., Qian Z.Q., Wang B. (2024). Novel Alzheimer’s disease subtypes based on functional brain connectivity in human connectome project. Sci. Rep..

[25] Scarapicchia V., Brown C., Mayo C., Jodie R., Gawryluk J.R. (2017). Functional Magnetic Resonance Imaging and Functional Near-Infrared Spectroscopy: Insights from Combined Recording Studies. Front. Hum. Neurosci..

[26] Darling A.L., Uversky V.N. (2018). Intrinsic disorder and posttranslational modifications: The darker side of the biological dark matter. Front. Genet..

[27] Uversky V.N. (2015). Intrinsically disordered proteins and their (disordered) proteomes in neurodegenerative disorders. Front. Aging Neurosci..

[28] Goedert M., Eisenberg D.S., Crowther R.A. (2017). Propagation of tau aggregates and neurodegeneration. Annu. Rev. Neurosci..

[29] Alquezar C., Arya S., Kao A.W. (2021). Tau Post-translational Modifications: Dynamic Transformers of Tau Function, Degradation, and Aggregation. Front. Neurol..

[30] Li B., Lu W., Chen Z. (2014). Regulation of Androgen Receptor by E3 Ubiquitin Ligases: For more or Less. Recept. Clin. Investig..

[31] Basheer N., Smolek T., Hassan I., Liu F., Iqbal K., Zilka N., Novak P. (2023). Does modulation of tau hyperphosphorylation represent a reasonable therapeutic strategy for Alzheimer’s disease? From preclinical studies to the clinical trials. Mol. Psychiatry.

[32] Congdon E.E., Sigurdsson E.M. (2018). Tau-targeting therapies for Alzheimer’s disease. Nat. Rev. Neurol..

[33] Goedert M., Spillantini M.G., Jakes R., Rutherford D., Crowther R.A. (1989). Multiple isoforms of human microtubule-associated protein tau: Sequences and localization in neurofibrillary tangles of Alzheimer’s disease. Neuron.

[34] Wegmann S., Biernat J., Mandelkow E.A. (2021). current view on Tau protein phosphorylation in Alzheimer’s disease. Curr. Opin. Neurobiol..

[35] Wojdała A.L., Bellomo G., Gaetani L., Teunissen C.E., Parnetti L., Chiasserini D. (2025). Immunoassay detection of multiphosphorylated tau proteoforms as cerebrospinal fluid and plasma Alzheimer’s disease biomarkers. Nat. Commun..

[36] Montoliu-Gaya L., Benedet A.L., Tissot C., Vrillon A., Ashton N.J., Brum W.S., Lantero-Rodriguez J., Stevenson J., Nilsson J., Sauer M. (2023). Mass spectrometric simultaneous quantification of tau species in plasma shows differential associations with amyloid and tau pathologies. Nat. Aging.

[37] Barthélemy N.R., Li Y., Joseph-Mathurin N., Gordon B.A., Hassenstab J., Benzinger T.L.S., Virginia Buckles V., Fagan A.M., Perrin R.J., Goate A.M. (2020). A soluble phosphorylated tau signature links tau, amyloid and the evolution of stages of dominantly inherited Alzheimer’s disease. Nat. Med..

[38] Ashton N.J., Pascoal T.A., Karikari T.K., Benedet A.L., Lantero-Rodriguez J., Brinkmalm G., Snellman A., Schöll M., Troakes C., Hye A. (2021). A new biomarker for incipient Alzheimer’s disease pathology. Acta Neuropathol..

[39] Bateman R.J., Xiong C., Benzinger T.L.S., Fagan A.M., Goate A., Fox N.C., Marcus D.S., Cairns N.J., Xie X., Blazey T.M. (2012). Clinical and biomarker changes in dominantly inherited Alzheimer’s disease. N. Engl. J. Med..

[40] Fischer I., Baas P.W. (2020). Resurrecting the mysteries of big tau. Trends Neurosci..

[41] Balmik A.A., Chinnathambi S. (2021). Methylation as a key regulator of Tau aggregation and neuronal health in Alzheimer’s disease. Cell Commun. Signal..

[42] Funk K.E., Thomas S.N., Schafer K.N., Cooper G.L., Liao Z., Clark D.J., Yang A.J., Kuret J. (2014). Lysine methylation is an endogenous post-translational modi- fication of tau protein in human brain and a modulator of aggregation propensity. Biochem. J..

[43] Morris M., Maeda S., Vossel K., Mucke L. (2011). The many faces of tau. Neuron.

[44] Wu G., Luo Y., Wang X. (2025). Conformation Pattern Changes in R1-Ps262 Tau Peptide Induced Endogenous Tau Aggregation, Synaptic Damage, and Cognitive Impairments. J. Alzheimers Dis..

[45] Morris M., Knudsen G.M., Maeda S., Trinidad J.C., Ioanoviciu A., Burlingame A.L., Mucke L. (2015). Tau post-translational modifications in wild-type and human amyloid precursor protein transgenic mice. Nat. Neurosci..

[46] Angioni D., Delrieu J., Hansson O., Fillit H., Aisen P., Cummings J., Sims J.R., Braunstein J.B., Sabbagh M., Bittner T. (2022). Blood Biomarkers from Research Use to Clinical Practice: What Must Be Done? A Report from the EU/US CTAD Task Force. J. Prev. Alzheimers Dis..

[47] Hansson O., Blennow K., Zetterberg H., Dage J. (2023). Blood biomarkers for Alzheimer’s disease in clinical practice and trials. Nat. Aging..

[48] Mielke M.M., Anderson M., Ashford J.W., Jeromin A., Lin P.J., Rosen A., Tyrone J., Vandevrede L., Willis D.R., Hansson O. (2024). Recommendations for clinical implementation of blood-based biomarkers for Alzheimer’s disease. Alzheimers Dement..

[49] Kirmess K.M., Meyer M.R., Holubasch M.S., Knapik S.S., Hu Y., Jackson E.N., Harpstrite S.E., Verghese P.B., West T., Fogelman I. (2021). The PrecivityAD™ test: Accurate and reliable LC-MS/MS assays for quantifying plasma amyloid beta 40 and 42 and apolipoprotein E proteotype for the assessment of brain amyloidosis. Clin. Chim. Acta..

[50] Schindler S.E., Bollinger J.G., Ovod V., Mawuenyega K.G., Li Y., Gordon B.A., Holtzman D.M., Morris J.C., Benzinger T.L.S., Xiong C. (2019). High-precision plasma β-amyloid 42/40 predicts current and future brain amyloidosis. Neurology.

[51] Palmqvist S., Janelidze S., Quiroz Y.T., Zetterberg H., Lopera F., Stomrud E., Su Y.I., Chen Y., Serrano G.E., Leuzy A. (2020). Discriminative accuracy of plasma phospho-tau217 for Alzheimer disease vs other neurodegenerative disorders. JAMA.

[52] Janelidze S., Bali D., Ashton N.J., Barthélemy N.R., Vanbrabant J., Stoops E., Vanmechelen E., He Y., Dolado A.O., Triana-Baltzer G. (2023). Head-to-head comparison of 10 plasma phospho-tau assays in prodromal Alzheimer’s disease. Brain.

[53] Brickman A.M., Manly J.J., Honig L.S., Sanchez D., Reyes-Dumeyer D., Lantigua R.A., Lao P.J., Stern Y., Vonsattel J.P., Teich A.F. (2021). Plasma p-tau181, p-tau217, and other blood-based Alzheimer’s disease biomarkers in a multi-ethnic, community study. Alzheimers Dement..

[54] Li Y., Schindler S.E., Bollinger J.G., Ovod V., Mawuenyega K.G., Weiner M.W., Shaw L.M., Masters C.L., Fowler C.J., Trojanowski J.Q. (2022). Validation of plasma amyloid-β 42/40 for detecting Alzheimer disease amyloid plaques. Neurology.

[55] Janelidze S., Teunissen C.E., Zetterberg H., Allué J.A., Sarasa L., Eichenlaub U., Bittner T., Ovod V., Verberk I.M.W., Toba K. (2021). Head-to-head comparison of 8plasma amyloid-β 42/40 assays in Alzheimer disease. JAMA Neurol..

[56] Villar-Piqué A., Schmitz M., Hermann P., Goebel S., Bunck T., Varges D., Ferrer I., Riggert J., Llorens F., Zerr I. (2019). Plasma YKL-40 in the spectrum of neurodegenerative dementia. J. Neuroinflamm..

[57] Craig-Schapiro R., Perrin R.J., Roe C.M., Xiong C., Carter D., Cairns N.J., Mintun M.A., Peskind E.R., Li G., Galasko D.R. (2010). YKL-40: A novel prognostic fluid biomarker for preclinical Alzheimer’s disease. Biol. Psychiatry.

[58] Wennström M., Surova Y., Hall S., Nilsson C., Minthon L., Hansson O., Nielsen H.M. (2015). The inflammatory marker YKL-40 is elevated in cerebrospinal fluid from patients with Alzheimer’s but not Parkinson’s disease or dementia with Lewy bodies. PLoS ONE.

[59] Pascual-Lucas M., Allué J.A., Sarasa L., Fandos N., Castillo S., Terencio J., Sarasa M., Tartari J.P., Sanabria Á., Tárraga L. (2023). Clinical performance of an antibody–free assay for plasma Aβ42/Aβ40 to detect early alterations of Alzheimer’s disease in individuals with subjective cognitive decline. Alzheimer’s Res. Ther..

[60] Vergallo A., Mégret L., Lista S., Cavedo E., Zetterberg H., Blennow K., Vanmechelen E., De Vos A., Habert M.-O., Potier M.-C. (2019). Plasma amyloid β 40/42 ratio predicts cerebral amyloidosis in cognitively normal individuals at risk for Alzheimer’s disease. Alzheimers Dement..

[61] Doecke J.D., Pérez-Grijalba V., Fandos N., Fowler C., Villemagne V.L., Masters C.L., Pesini P., Manuel Sarasa M., AIBL Research Group (2020). Total Aβ42/Aβ40 ratio in plasma predicts amyloid-PET status, independent of clinical AD diagnosis. Neurology.

[62] Sugarman M.A., Zetterberg H., Blennow K., Tripodis Y., Ann C., McKee A.C., Stein T.D., Brett Martin B., Palmisano J.N., Eric G. (2020). A longitudinal examination of plasma neurofilament light and total tau for the clinical detection and monitoring of Alzheimer’s disease. Neurobiol. Aging..

[63] Shir D., Graff-Radford J., Hofrennin E.I., Lesnick T.G., Przybelski S.A., Lowe V.J., David S., Knopman D.S., Ronald C., Petersen R.C. (2022). Association of plasma glial fibrillary acidic protein (GFAP) with neuroimaging of Alzheimer’s disease and vascular pathology. Alzheimers Dement..

[64] Chatterjee P., Vermunt L., Gordon B.A., Pedrini S., Boonkamp L., Armstrong N.J., Xiong C., Singh A.K., Li Y., Sohrabi H.R. (2021). Plasma glial fibrillary acidic protein is elevated in cognitively normal older adults at risk of Alzheimer’s disease. Transl. Psychiatry.

[65] Shen X.-N., Huang S.-Y., Cui M., Zhao Q.-H., Guo Y., Huang Y.-Y., Zhang W., Ma Y.-H., Chen S.-D., Zhang Y. (2023). Plasma glial fibrillary acidic protein in the Alzheimer disease continuum: Relationship to other biomarkers, differential diagnosis, and prediction of clinical progression. Clin. Chem..

[66] Milà-Alomà M., Ashton N.J., Shekari M., Salvadó G., -Romero P., Montoliu-Gaya L., Andrea L., Benedet A.L., Karikari T.K., Juan Lantero-Rodriguez J. (2022). Plasma p-tau 231 and p-tau 217 as state markers of amyloid-β pathology in preclinical Alzheimer’s disease. Nat. Med..

[67] Suárez-Calvet M., Karikari T., Ashton N., Rodríguez L., Milà-Alomà M., Juan Domingo Gispert J.D., Gemma Salvadó G., Carolina Minguillon C., Karine Fauria K., Shekari M. (2020). Novel tau biomarkers phosphorylated at T181, T217 or T231 rise in the initial stages of the preclinical Alzheimer’s continuum when only subtle changes in Aβ pathology are detected. EMBO Mol. Med..

[68] Ashton N.J., Puig-Pijoan A., Milà-Alomà M., Aida Fernández-Lebrero A., Greta García-Escobar G., González-Ortiz F., Kac P.R., Brum W.S., Andréa L., Benedet A.L. (2023). Plasma and CSF biomarkers in a memory clinic: Head-to-head comparison of phosphorylated tau immunoassays. Alzheimers Dement..

[69] Pais M.V., Forlenza O.V., Diniz B.S. (2023). Plasma Biomarkers of Alzheimer’s Disease: A Review of Available Assays, Recent Developments, and Implications for Clinical Practice. J. Alzheimers Dis. Rep..

[70] Cano A., Capdevila M., Puerta R., Arranz J., Montrreal L., de Rojas I., García-González P., Olivé C., García-Gutiérrez F., Sotolongo-Grau O. (2024). Clinical value of plasma pTau181 to predict Alzheimer’s disease pathology in a large real-world cohort of a memory clinic. Ebiomedicine.

[71] Baiardi S., Quadalti C., Mammana A., Dellavalle S., Zenesini C., Sambati L., Pantieri R., Polischi B., Romano L., Matteo Suffritti M. (2022). Diagnostic value of plasma p-tau 181, NfL, and GFAP in a clinical setting cohort of prevalent neurodegenerative dementias. Alzheimer’s Res. Ther..

[72] Karikari T.K., Ashton N.J., Brinkmalm G., Brum W.S., Benedet A.L., Montoliu-Gaya L., Lantero-Rodriguez J., Pascoal T.A., Suárez-Calvet M., Rosa-Neto P. (2022). Blood phospho-tau in Alzheimer disease: Analysis, interpretation, and clinical utility. Nat. Rev. Neurol..

[73] Rosenberg A., Öhlund-Wistbacka U., Hall A., Bonnard A., Göran Hagman G., Rydén M., Thunborg C., Wiggenraad F., Sandebring-Matton A., Solomon A. (2022). β-Amyloid, Tau, Neurodegeneration Classification and Eligibility for Anti-amyloid Treatment in a Memory Clinic Population. Neurology.

[74] Dammer E.B., Hantaraman A., Ping L., Duong D.M., Modeste E.S., Ravindran S.P., Gudmundsdottir V., Fricke A., Gomez G.T., Walker K.A. (2024). Proteomic analysis of Alzheimer’s disease cerebrospinal fluid reveals alterations associated with APOE ε4 and atomoxetine treatment. Sci. Transl. Med..

[75] Dammer E.B., Ping L., Duong D.M., Erica S., Modeste E.S., Nicholas T., Seyfried N.T., Lah J.J., Levey A.I., Erik C.B. (2022). Multi-platform proteomic analysis of Alzheimer’s disease cerebrospinal fluid and plasma reveals network biomarkers associated with proteostasis and the matrisome. Alzheimer’s Res. Ther..

[76] Mielke M.M., Frank R.D., Dage J.L., Jeromin A., Ashton N.J., Blennow K., Karikari T.K., Vanmechelen E., Zetterberg H., Algeciras-Schimnich A. (2021). Comparison of Plasma Phosphorylated Tau Species with Amyloid and Tau Positron Emission Tomography Neurodegeneration, Vascular Pathology, and Cognitive Outcomes. JAMA Neurol..

[77] Bayoumy S., Verberk I.M.W., den Dulk B., Hussainali Z., Zwan M., van der Flier W.M., Ashton N.J., Zetterberg H., Blennow K., Vanbrabant J. (2021). Clinical and analytical comparison of six Simoa assays for plasma P-tau isoforms P-tau181, P-tau217, and P-tauAlzheimers. Res. Ther..

[78] Barthélemy N.R., Horie K., Sato C., Bateman R.J. (2020). Blood plasma phosphorylated tau isoforms track CNS change in Alzheimer’s disease. J. Exp. Med. Nov..

[79] Chen S.-D., Huang Y.-Y., Shen X.-N., Guo Y., Ta L., Dong Q., Yu J.-T. (2021). Longitudinal plasma phosphorylated tau 181 tracks disease progression in Alzheimer’s disease. Transl. Psychiatry.

[80] Janelidze S., Berron D., Smith R., Strandberg O., Nicholas K., Proctor N.K., Dage J.L., Stomrud E., Palmqvist S., Mattsson-Carlgren N. (2021). Associations of Plasma Phospho-Tau217 Levels with Tau Positron Emission Tomography in Early Alzheimer Diseaseansson. JAMA Neurol..

[81] Rodriguez J.L., Karikari T.K., Suárez-Calvet M., Troake C., King A., Emersic A., Aarsland D., Hye A., Henrik Zetterberg H., Blennow K. (2020). Plasma p-tau181 accurately predicts Alzheimer’s disease pathology at least 8 years prior to post-mortem and improves the clinical characterization of cognitive decline. Acta Neuropathol..

[82] Korecka M., Shaw L.M. (2021). Mass spectrometry-based methods for robust measurement of Alzheimer’s disease biomarkers in biological fluids. J. Neurochem..

[83] Yi L., Wenbin Liu W., Wang Z., Ren D., Peng W. (2017). Characterizing Alzheimer’s disease through metabolomics and investigating anti-Alzheimer’s disease effects of natural products. Ann. N. Y. Acad. Sci..

[84] Spellman D.S., Wildsmith K.R., Honigberg L.A., Tuefferd M., Baker D., Raghavan N. (2015). Development and evaluation of a multiplexed mass spectrometry-based assay for measuring candidate peptide biomarkers in Alzheimer’s Disease Neuroimaging Initiative (ADNI) CSF. Proteom. Clin. Appl..

[85] Pomilio A.B., Vitale A.A., Lazarowski A.J. (2022). Neuroproteomics Chip-Based Mass Spectrometry and Other Techniques for Alzheimer’s Disease Biomarkers–Update. Curr. Pharm. Des..

[86] Pottiez G., Yang L., Stewart T., Song N., Aro P., Galasko D.R., Quinn J.F., Peskind E.R., Shi M., Zhang J. (2017). Mass Spectrometry-Based Method to Quantify in Parallel Tau and Amyloid β 1–42 in CSF for the Diagnosis of Alzheimer’s Disease. J. Proteome Res..

